# West Nile Virus and High Death Rate in American Crows

**DOI:** 10.3201/eid1004.030499

**Published:** 2004-04

**Authors:** Sarah A. Yaremych, Richard E. Warner, Phil C. Mankin, Jeff D. Brawn, Arlo Raim, Robert Novak

**Affiliations:** * University of Illinois, Urbana, Illinois, USA; † Illinois Natural History Survey, Champaign, Illinois, USA

**Keywords:** American Crow, West Nile virus, flavivirus, avian diseases, mortality, reverse transcriptase–polymerase chain reaction, immunohistochemistry, blocking ELISA

## Abstract

We document effects of West Nile virus (WNV) on American Crows. More than two thirds of our crows died of WNV infection, peaking when the proportion of infected mosquitoes at roosts was greatest. WNV antibody prevalence in crows was low. Local ecologic effects can be dramatic as WNV inhabits new areas.

The introduction of West Nile virus (WNV) to North America in the summer of 1999 prompted concern about effects of WNV disease in wildlife. Though the disease has spread rapidly since its introduction, little documentation is available describing the effect of WNV on free-ranging wildlife species. We monitored the emergence and prevalence of WNV infection in American Crows (*Corvus brachyrhynchos*) in Illinois during the spring and summer of 2002; relative to other states, Illinois had the largest number of human West Nile meningoencephalitis cases in 2002 (*1*).

## The Study

Beginning in February 2002, we captured 156 American Crows with Australian crow traps (*2*) in Champaign/Urbana in east-central Illinois. Each captured crow was banded, aged by palate coloration (*3*), measured for sex determination by discriminant function analysis (*4*), and painted across the span of the tail feathers for identification. Blood samples were taken from all crows to detect antibodies to WNV antigen. Radio transmitters were attached to a subsample of crows. Transmitters were custom designed and weighed <2 g for tail-mount and <3 g for collar-mount, with an expected battery life of 6 months. A tail-mount attachment was used most frequently, modifying the method of Dunstan (*5*), and individually sized collar-mount transmitters (*6*) were attached during the molting season. Each crow with a transmitter was tracked at least 10 times per week by vehicle equipped with a receiving system, and locations were stratified by time of day (morning, afternoon, or night). Birds that could not be located due to transmitter failure, loss, or other reason were searched for with fixed-wing aircraft. This intensive telemetry allowed us to locate dead crows soon after death.

During the months of May through October, radio transmitters were attached to 39 crows. This cohort consisted of 5 adults, 6 sub-adults, and 28 hatch-year crows; 9 were male and 30 were female, as determined by discriminant function analysis or by gonadal observation on dead crows. Of these crows, the fates of 11 birds could not be determined because of transmitter loss, failure, or disappearance. The fates of the remaining 28 birds, comprising 22 females and 6 males, were monitored until death or for the duration of the study (through October). Of the remaining 28 crows, 19 were recovered dead and confirmed positive for WNV with immunohistochemistry (*7*) or TaqMan reverse transcriptase–polymerase chain reaction (*8*) yielding a 68% (95% confidence interval [CI] 48% to 84%) WNV-attributed population death rate. The 19 dead crows consisted of 15 females and 4 males; rate of death from WNV did not differ between sexes (χ^2^ = 0.0015, df = 1, p = 0.97), or age classes (Mann-Whitney U = 263, df = 1, p = 0.48). Two crows died and tested negative for WNV during the 6-month observation period of May through October, and 7 birds survived the observation period.

Since our 6-month observation period covered the complete WNV transmission season, no additional WNV deaths would be expected when extrapolating these data to represent a full year. If we assume the number of non-WNV deaths associated with our 6-month study would double in a full year, we extrapolate that the annual death rate for the cohort of 28 crows includes 19 WNV deaths and 4 non-WNV deaths. Therefore, we calculate the annual survival rate of American Crows in east-central Illinois to be 17.9%. This estimate is conservative, as more crows would be expected to die of non-WNV causes in the winter months due to harsher living conditions. The average annual survival rate for breeding-age American Crows from six studies across North America has been estimated at 89.6% (*9*), though comparing survival rates between studies is difficult as our sample was largely biased towards hatch-year crows.

We also monitored the prevalence of WNV infection in mosquitoes at roost sites associated with our radio-tracked crows. Crows were tracked to six nighttime roost sites continually used throughout the summer. Mosquitoes were collected weekly at each of these sites for 15 weeks using both CO_2_-baited light traps and gravid traps, for a total of 90 trap-nights with each trap type. Throughout the season, we collected 595 pools of mosquitoes representing 10 species, including *Culex (culex)* spp., *Aedes vexans*, *Anopheles punctipennis*, *A. quadrimaculatus*, *Ochlerotatus triseriatus*, *O. trivittatus*, *Uranotaenia sapphirina*, *Coquillettidia perturbans*, *Orthopodomyia signifera*, and ***Culiseta inornata*.** A pool refers to a uniform grouping of 1–50 mosquitoes collected on the same day at the same location, sorted by species and sex for analysis.

Twenty pools were WNV-positive, with the first positive pool collected the week of July 19, and the last positive pool collected the week of September 20. Of the 20 positive pools, 18 were female and 1 was male *Culex (culex)* spp. and 1 was female *Anopheles punctipennis*. We collected 14 of the positive pools in gravid traps and 6 in CO_2_-baited light traps; positive pools were found at all six roost sites. Combined species minimum infection rates (MIRs) per 1,000 mosquitoes were calculated by week and ranged from 0 to 19. The lowest weekly survival rate of crows occurred from August 16 to September 6 and coincided with the highest MIR in the sampled mosquitoes (Figure).

**Figure:**
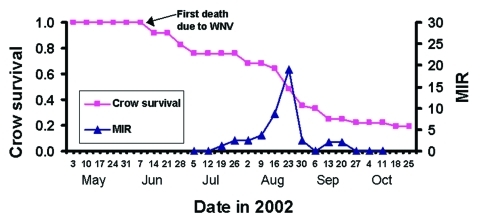
Survival curve (Kaplan-Meier curve; staggered-entry method) (*10*,*11*) for radio-tracked American Crows (N = 39) relative to the weekly minimum infection rates (MIR) of mosquitoes collected by week at radio-tracked crow roost sites in east-central Illinois in 2002.

To estimate survival in WNV-exposed crows, we used a blocking enzyme-linked immunosorbent assay (*12*) to test blood samples (N = 156) from all captured crows collected from late February through October in Champaign/Urbana, including the radio-tracked sample. The sample included 13 adults, 13 sub-adults, and 130 hatch-year crows. An inhibition value of ≥30% of monoclonal antibody 3.1112G was required to indicate the presence of WNV-specific antibodies. Inhibition of ≥30% of monoclonal antibody 2B2 served as a confirmatory test, although this antibody is not specific for WNV. Blitvich et al. (*12*) determined that the most efficient assays for detecting WNV serum antibodies were those that used monoclonal antibodies (MAbs) 3.1112G and 2B2 and that the use of MAb 3.1112G can differentiate between St. Louis encephalitis virus and WNV infections. Serology showed that WNV-specific antibodies were present in 5 of 156 free-ranging crows (Table). One of these seropositive crows was tracked and found dead and positive for WNV 56 days after the blood sample was taken.

**Table:** Details of the five seropositive crows captured in east-central Illinois, 2002^a^

ID	Bleeding date	Age class	Fate
108	26 April	Adult	Radio-tracked, molted transmitter 19 June
117	5 June	Adult	Radio-tracked, died and negative for WNV 18 June
130	9 July	Hatch-year	Radio-tracked, dead and positive for WNV 3 September
180	1 August	Hatch-year	Not radio-tracked
228	30 August	Hatch-year	Not radio-tracked

^a^WNV, West Nile virus

## Conclusions

This study represents the first peer-reviewed publication describing the death rate from WNV in a tracked wild bird population. While not all infected crows succumbed to WNV, American Crows and other corvids appear to be more differentially susceptible to death due to WNV than noncorvids (*13*–*15*). After the arrival of WNV to new areas, crows experience high death rates (*16*). The relationship between total observed death rate and WNV-attributed death rate in our study indicates a very high level of involvement of WNV in American Crow deaths (90.5%). The proportion of crow deaths attributed to WNV in our study was different from that in other studies in other locations and years. For example, during the 2000 transmission season in the state of New York, 47% of 1,687 dead American Crows tested positive for WNV, with 67% of crows within the epicenter testing positive (*15*). Surveillance data suggest that the involvement of WNV in the deaths of noncorvid species submitted for WNV testing is much less than involvement of WNV in the deaths of crows (<40% in the 2000 New York study [*15*]).

Our observation of a seropositive crow that died and tested positive for WNV <2 months after the blood sample was taken merits attention. Similarly, a Red-tailed Hawk that died in the middle of winter raised questions whether the virus could have been acquired earlier, with latent infection later causing death (*17*). Further investigation of arboviral recrudescence is necessary. Values of MAb inhibition in this crow were just above the threshold for considering a sample positive for WNV-specific antibodies, suggesting a weak response. The antibodies may be due to passive immunity transferred by a parent; however, this crow was around 2.5 months old, and the duration of maternal antibodies may not last this long. The duration of maternal antibodies in crows has yet to be studied.

Experimentally derived death rates of American Crows infected with WNV were 100% in two studies (*13*,*14*), in which 8 and 10 infected crows died within 6 and 7 days postinfection, respectively. These studies, combined with our findings, raise concerns about the potential effect of WNV on threatened or endangered corvids, including the Florida Scrub-Jay (*Aphelocoma coerulescens*), the Hawaiian Crow (*Corvus hawaiiensis*), and the Mariana Crow (*Corvus kubaryi*). As globalization increases and exotic pathogens continue to be introduced, native species will experience new selective pressures with unknown ecologic consequences.
